# *Leishmania infantum* Asparagine Synthetase A Is Dispensable for Parasites Survival and Infectivity

**DOI:** 10.1371/journal.pntd.0004365

**Published:** 2016-01-15

**Authors:** Joana Faria, Inês Loureiro, Nuno Santarém, Sandra Macedo-Ribeiro, Joana Tavares, Anabela Cordeiro-da-Silva

**Affiliations:** 1 Parasite Disease Group, Instituto de Biologia Molecular e Celular da Universidade do Porto, Porto, Portugal; 2 Instituto de Investigação e Inovação em Saúde, Universidade do Porto, Porto, Portugal; 3 Protein Crystallography Group, Instituto de Biologia Molecular e Celular da Universidade do Porto, Porto, Portugal; 4 Departamento de Ciências Biológicas, Faculdade de Farmácia, Universidade do Porto, Porto, Portugal; Northeastern University, UNITED STATES

## Abstract

A growing interest in asparagine (Asn) metabolism has currently been observed in cancer and infection fields. Asparagine synthetase (AS) is responsible for the conversion of aspartate into Asn in an ATP-dependent manner, using ammonia or glutamine as a nitrogen source. There are two structurally distinct AS: the strictly ammonia dependent, type A, and the type B, which preferably uses glutamine. Absent in humans and present in trypanosomatids, AS-A was worthy of exploring as a potential drug target candidate. Appealingly, it was reported that AS-A was essential in *Leishmania donovani*, making it a promising drug target. In the work herein we demonstrate that *Leishmania infantum* AS-A, similarly to *Trypanosoma* spp. and *L*. *donovani*, is able to use both ammonia and glutamine as nitrogen donors. Moreover, we have successfully generated *LiASA* null mutants by targeted gene replacement in *L*. *infantum*, and these parasites do not display any significant growth or infectivity defect. Indeed, a severe impairment of *in vitro* growth was only observed when null mutants were cultured in asparagine limiting conditions. Altogether our results demonstrate that despite being important under asparagine limitation, *Li*AS-A is not essential for parasite survival, growth or infectivity in normal *in vitro* and *in vivo* conditions. Therefore we exclude AS-A as a suitable drug target against *L*. *infantum* parasites.

## Author Summary

It was recently described that asparagine synthetase A (AS-A) of trypanosomatids uses not only ammonia but also glutamine for asparagine formation, which was a surprising feature for a type A AS. Interestingly, *Leishmania donovani* AS-A was reported to be essential for parasite survival, and once a human homologue was absent, this enzyme emerged as a novel drug target candidate. *Leishmania infantum* encodes for a functional AS-A enzyme, which also uses either ammonia or glutamine as nitrogen donor for asparagine synthesis. In *L*. *infantum*, *ASA* ablation drives parasites auxotrophic to asparagine, however, *Li*AS-A is not detrimental for parasite survival, growth or infectivity. AS-A is therefore unlikely to be a suitable drug target against *Leishmania* parasites.

## Introduction

Leishmaniasis is a vector borne human disease, caused by several species of digenetic protozoan parasites belonging to genus *Leishmania*. The clinical presentations of this neglected tropical disease vary from selfhealing cutaneous manifestations to potentially fatal, if untreated, visceral ailment [1]. The most severe form of the disease, designated as visceral leishmaniasis (VL) is mainly associated to *Leishmania donovani* or *Leishmania infantum*. Due to the absence of human vaccines, VL control relies mainly on chemotherapy and appropriate vector control [2]. The traditional therapeutic options are associated with significant limitations (cost, toxicity, complex administration regimes, resistance) averting disease control in endemic areas [3]. As consequence, according to World Health Organization between 20,000 and 30,000 people (mostly children) die every year, rendering the search for novel chemotherapeutic options a priority [4].

Asparagine (Asn) metabolism has been under the spotlight in the recent years. Asn is the last nonessential amino acid to be synthesised from glucose metabolism [5]. For many years it seemed it was not involved in any other pathway but protein synthesis in mammalian cells, contrasting with the other 19 common amino acids [6]. Nonetheless, several recent studies suggest Asn somehow coordinates cell responses with metabolic reserves and ultimately regulates cell fate [5]. In many pathogenic microorganisms, functional studies on L-asparaginase and Asn transporters have implicated Asn metabolism in survival, invasion and/or virulence [7–14].

Asparagine synthetase (AS) is another key player in Asn metabolism, it catalyses Asn formation from aspartate in an ATP dependent manner using ammonia or glutamine as nitrogen donors. The reaction mechanism comprises two crucial steps: 1) the formation of β-aspartylAMP, in which β-carboxylate group of aspartate is activated by ATP; 2) nucleophilic attack by an ammonium ion. This mechanism mirrors the close evolutionary relation to aminoacyl-tRNA synthetase enzymes [15]. There are two structurally distinct types of AS: A and B [16]. Type B (AS-B, EC. 6.3.5.4) uses preferably glutamine over ammonia and can be found in prokaryotes and eukaryotes (mammalian cells, yeasts, *Chlamydomonas reinhardtii*, higher plants) [17–23]. Type A (AS-A, EC. 6.3.1.1) are found mainly in prokaryotes (*Escherichia coli* [24] and *Klebsiella aerogenes* [25]) or in archaea (*Pyrococcus abyssi* [26]) and described as strictly ammonia dependent. Surprisingly, kinetoplastids and other protozoans, despite being eukaryotes, possess not only a putative AS-B but also a bacterial type AS-A [27–29]. Moreover AS-A from *Trypanosoma brucei*, *Trypanosoma cruzi* [28] and *L*. *donovani* [29] parasites were reported to use glutamine as nitrogen donor as well.

Several roles have been associated to bacterial AS. For instance, in *Pasteurella multocida*, AS-A is significantly upregulated during host infection, in *Mycobacterium smegmatis* AS-B is involved in natural resistance to antibiotics and in *Mycobacterium tuberculosis*, AS-B was reported to be required for *in vitro* growth [30–33].

Recently our group showed that in *T*. *brucei* bloodstream forms AS-A knockdown has no impact on parasites growth or infectivity, except upon Asn deprivation. These results suggest Asn main sources are AS-A mediated synthesis and extracellular uptake [28]. Surprisingly, in *L*. *donovani*, AS-A was claimed to be essential for parasites survival and emerged as a promising drug target due to the absence of a human homologue [29]. Additionally, these results also suggest that Asn homeostasis could be differently regulated among trypanosomatids. These parasites present different amino acid requirements for either energetic or osmotic functions in different stages of their life cycles and as a reflex of the different environmental stimuli they receive in the vector or mammalian host [34]. Across trypanosomatids’ species, the amino acid transporters (AAT) repertoire has a high interspecific variation, regarding number, affinity, specificity and capacity [34]. For instance, in the case of cysteine, a crucial amino acid for thiol biosynthesis, *Leishmania major* contrarily to *T*. *brucei*, fails to uptake it at a rate that ensures the intracellular pool is enough for optimal growth. Therefore, these parasites rely mainly on pathways that enable cysteine synthesis [35].

In this work, we have biochemically characterized *L*. *infantum* AS-A (*Li*AS-A), and to gain further insights on AS-A essentiality across different *Leishmania* species, we have performed gene replacement studies in *L*. *infantum*.

## Methods

### Ethics statement

All experiments were carried out in accordance with the IBMC.INEB Animal Ethics Committee and the Portuguese National Authority for Animal Health (DGAV) guidelines, according to the statements on the directive 2010/63/EU of the European Parliament and of the Council. DGAV approved the animal experimentation presented in this manuscript under the license DGAV number 25268/2013-10-02.

### Chemicals and reagents

L-asparagine, L-aspartic acid sodium salt monohydrate, L-glutamine, L-glutamatic acid salt hydrate, ATP disodium salt hydrate, AMP disodium salt, sodium pyrophosphate decahydrate, ninhydrin, dNTPs, ammonium chloride, magnesium chloride, tween-20, tris-base, urea, thiourea, DTT, triton X-100 and IPTG (isopropyl-β-D- thiogalactopyranoside) were purchased from Sigma. Oligonucleotide primers were obtained from STAB VIDA. Restriction endonucleases were from New England Biolabs. Polyclonal antibodies against *Li*AS-A were obtained in rabbits inoculated with purified recombinant His-tagged *Li*AS-A. *E*. *coli* L-asparaginase was purchased from Prospec.

### Parasites

*L*. *infantum* (MHOM/MA/67/ITMAP-263) promastigote forms were grown at 26°C in complete RPMI 1640 medium [36]. For *in vitro* and *in vivo* characterization, different cell lines were firstly recovered from the spleen of infected BALB/c to restore virulence, and subsequently maintained in culture no longer than 10 passages [36]. Axenic amastigotes were grown in MAA complete medium [36], at 37°C, 5% CO_2_. Depending on the analysis, protein extracts were prepared as follows: 1) 1 x 10^7^ late-stationary promastigotes were resuspended in T8 lysis buffer (tris-base 0.6%, urea 42%, thiourea 15%, DTT 0.3%, triton X-100 1%); or 2) 1 x 10^8^ promastigotes or axenic amastigotes were resuspended in 100 μL of PBS containing protease inhibitor (Roche) and following 6 freezing/thaw cycles, the parasite suspensions supernatants were recovered and then quantified using Bio-Rad DC Protein Assay (Biorad).

### AS-A protein alignments and *Li*AS-A/*Lm*AS-A homology models

*Ec*AS-A, *Li*AS-A, *Lm*AS-A, *Tb*AS-A and *Tc*AS-A protein alignments were performed using the ClustalW program [37]. Aline program, *Version 011208* [38], was used for editing protein sequence alignments. *Li*AS-A and *Lm*AS-A homology models were obtained with SWISS-MODEL, using *Ec*AS-A crystal structure (Protein Data Bank (PDB) 12AS [15]) as a template (percentage of sequence identity of ~50–60% in both cases) [39–41]. The 3D models were illustrated using PyMOL program (The PyMOL Molecular Graphics System, Version 1.3, Schrödinger, LLC).

### Cloning *ASA* genes

Asparagine synthetase A (*ASA*) from *L*. *infantum* (LinJ.26.0790; chromosome LinJ.26; 234298–235360) was obtained by performing PCR on genomic DNA, extracted using DNAzol (Invitrogen) [42–44], using primers 1 + 2 (S1 Table). PCR conditions were as follows: initial denaturation (2 min at 94°C), 35 cycles of denaturation (30 s at 94°C), annealing (30 s at 50°C) elongation (2 min at 68°C) and a final extension step (10 min at 68°C). Another restriction strategy was required to clone the gene into a *Leishmania* overexpression vector–pSPα*BLAST*α, and the sequence was amplified using primers 3 + 4 (S1 Table). PCR conditions were as follows: initial denaturation (2 min at 94°C), 30 cycles of denaturation (15 s at 94°C), annealing (30 s at 55°C) elongation (1 min at 72°C) and a final extension step (10 min at 72°C). All PCR products were cloned into a pGEM-T Easy vector (Promega) and sent for sequencing.

### Expression and purification of poly-His-tagged recombinant *Li*AS-A

The *LiASA* gene was excised from the pGEM-T Easy vector (using NdeI/EcoRI), and subcloned into pET28a(+) expression vector (Novagen). The resulting construct presented a poly-His tag (6x Histine residues) at the N-terminal and was transformed into *E*. *coli* BL21DE3. The recombinant protein was expressed by induction of log-phase cultures with 0.5 mM of IPTG at 18°C O/N. Bacteria were harvested and resuspended in buffer A (0.5 M NaCl, 20 mM Tris.HCl, pH 7.6). The sample was sonicated, according to the following conditions: output 4, duty cycle 50%, 10 cycles with 15 s each (Branson sonifier 250), followed by centrifugation to obtain the bacterial crude extract. For enzymatic activity experiments and rabbit polyclonal antibody production, the recombinant enzyme was purified in one step using Ni^2+^ resin (Qiagen) pre-equilibrated in buffer A. The column was washed sequentially with buffer A, bacterial crude extract, and buffer A with increasing concentrations of imidazole. *Li*AS-A was eluted in the fractions of buffer A containing 100 to 500 mM of imidazole. Dialysis was performed against PBS.

For additional activity tests, oligomeric form and Stokes’ radius assessment, a deeper purification was performed. Firstly, the enzyme was purified by affinity chromatography, using a Histrap HP column (GE Healthcare), charged with nickel sulphate and equilibrated in buffer A, and posteriorly mounted in an AKTAPrimer Plus (GE Healthcare) system, at 4°C. Secondly, it was purified by size exclusion chromatography, in a Hiprep 26/60 Sephacryl S-200 column (GE Healthcare), previously equilibrated with running buffer (150 mM NaCl, 20 mM Tris, pH 7.6). The last purification step was a preparative ion exchange chromatography, using an UNO Q-1 (Bio-Rad, Cat. No 720–0001) column, mounted in a BioLogic DuoFlow (Bio-Rad) device, at 4°C. The fractions were finally analysed by analytic size exclusion chromatography and analytic ion exchange chromatography, using AktaPurifier10 system (GE Healthcare), using Superose 12 10/300GL (GE Healthcare) column and a UNO Q-1 (Bio-Rad, Cat. No 720–0001) column, respectively. The final fractions were concentrated using Millipore centrifugal filter 30K (Amicon Ultra).

Concentration was determined measuring the absorbance at 280 nm using the theoretical molar extinction coefficient of 46910 M^-1^.cm^-1^ for *Li*AS-A, making use of NanoDrop ND-1000 Spectrophotometer (NanoDrop Technologies). The purified recombinant protein was resolved in SDS/PAGE and stained with Coomassie Brilliant Blue G-250 (Biorad).

For estimation of the *Li*AS-A oligomeric state the purified recombinant protein was analysed by analytic size exclusion chromatography, using the above described conditions. Blue dextran (2,000 kDa), catalase (MW 232 kDa, Stokes radius (SR) 5.22 nm), aldolase (MW 158 kDa, SR 4.81 nm), albumin (MW 67 kDa, SR 3.55 nm), ovalbumin (MW 43 kDa, SR 3.05 nm), chymotrypsinogen A (MW 25 kDa, SR 2.09 nm) and ribonuclease (MW 13.7 kDa, SR 1.64 nm) were used as standards. A calibration curve relating Log (MW) or Log(SR) with K_av_ was performed (K_av_ is (V_e_-V_0_)/(V_t_-V_0_), in which V_e_ is elution volume, V_0_ is the exclusion volume given by blue dextran and V_t_ is the total volume of the column).

### Differential scanning fluorimetry

In a 96-well, thin-walled white PCR plate, 5 μl of *Li*AS-A (2.4 μM) were mixed with 5 μl of 10x SYPRO Orange (λ_exc_ 485 nm; λ_em_ 625 nm) and 40 μl of water or the ligands and ligands combinations to be tested. Plates were then sealed and placed into a BioRad iCycler5 PCR instrument. Measurements were taken every minute in 0.5°C increments from 25° to 95°C. Subsequent analysis of the fluorescent data using Biorad iCycler iQ Optical System *Software* Version 3.1 yielded the protein melting temperature (T_m_) for *Li*AS-A.

### Western-blot analysis

Western-blot was performed aiming different purposes: (1) His-tag labelling of recombinant proteins, (2) *Li*AS-A labelling in total soluble parasite extracts to assess protein expression throughout the life cycle, (3) *Li*AS-A labelling in mutants and (4) to assess protein distribution upon digitonin fractionation. One μg of recombinant *Li*AS-A, 20 μg of total soluble extracts from both promastigote and amastigote forms, or 1 x 10^7^ parasites were resolved in SDS-PAGE and transferred onto a nitrocellulose membrane (TransBlot Turbo, Bio-Rad), which was blocked, probed, washed and developed as previously described [28]. The following primary antibodies were used: rabbit anti-His-tag (MicroMol-413, 1:1000), mouse anti-α-tubulin (clone DM1A, Neomarkers, 1:1000), rabbit anti-*Li*AS-A (1:1000), rabbit anti-*Li*CS (cysteine synthase, 1:2000), rabbit anti-*Ld*HGPRT (hypoxanthine guanine phosphoribosyl transferase, 1:2000), and rabbit anti-*Tb*Enolase (1:5000). *Horseradish* peroxidase-conjugated goat anti-rabbit or goat anti-mouse IgG (Amersham) (1:5000 for 1 h, at RT) were used as the secondary antibody. ImageJ software (version 1.43u) was used for protein semi-quantification.

### Enzymatic assay

A 150 μl enzymatic mixture containing 85 mM Tris.HCl, 8.4 mM magnesium and varying concentrations of aspartate, ammonia and ATP was assayed. The assay was performed as previously described [28], and ultimately absorbance at 340 nm was measured [45]. To determine the optimal conditions for kinetic parameters determination, reaction linearity was checked by varying enzyme concentration and time. The final reaction conditions used 7.5 μg of enzyme per assay and 15 min incubation at 37°C. A pH range of 7.0 to 9.0 was assessed, and pH 7.6 was selected as the optimal one to perform the following enzyme assays. To determine the *K*_m_ of each substrate a certain range of concentration was used and the remaining substrates were maintained in excess. For aspartate, ammonia, ATP and glutamine, the following concentrations were used: 1.25 to 20, 0.78 to 50, 0.625 to 10 and 1.56 to 25 mM, respectively.

### Generation of *LiASA* null mutants

A targeted gene replacement strategy was used for *L*. *infantum ASA* gene knock-out. Briefly, *ASA* flanking regions were amplified from *L*. *infantum* genomic DNA and were linked to neomycin phosphotransferase (*NEO*) or hygromycin phosphotransferase (*HYG*) genes using a fusion PCR approach. The 5’ and 3’ UTR were amplified using primers 1 + 2 and 3 + 4 (S2 Table), respectively. *NEO* and *HY*G were amplified from pSP72*αNEOα* and pGL345*HYG* templates, using primers 5 + 6 and 7 + 8 (S2 Table) respectively, which possess around 30 nucleotides of the 5’ UTR in the sense primer and the first 30 nucleotides of the 3’ UTR in the antisense primer. 5’ UTR_*NEO_*3’ UTR and 5’ UTR_*HYG*_3’ UTR constructs were obtained using primers 1 + 4 (S2 Table). Mid-log promastigotes were transfected with approximately 10 μg of linear construct, obtained by fusion PCR, using an AMAXA Nucleofector II device with Human T-cell nucleofector kit (Lonza). The day after transfection drug selection was carried out at 20 μg/mL of G418 (Invitrogen) and 50 μg/mL of hygromycin B (InVivoGen). Parasite cloning was performed by diluting the parasite suspension to a concentration of 0.5 cells/well, using SDM culture medium. The drug concentrations for clone maintenance correspond to half of the selection concentrations.

### Generation of *LiASA* overexpressor (OE) and null mutants’ complementation

*LiASA* gene was excised from the pGEM-T Easy vector (using XbaI/NdeI) and subcloned into pSP72*αBLASTα* vector. Mid-log promastigotes, WT and dKO mutants, were transfected with approximately 10 μg of plasmid DNA as above in order to generate an overexpressing line (OE) or complemented null mutants, respectively. Drug selection was carried out at 30 μg/ml of blasticidin (InVivoGen).

### PCR and Southern-blot analysis of *LiASA* mutants

*LiASA* mutants were analysed by PCR using Taq polymerase (NZYTech) for the following events: *LiASA* presence; *NEO* 5’ integration; *NEO* 3’ integration; *HYG* 5’ integration and *HYG* 3’ integration, using primers pairs 9 + 10, 11 + 12, 13 + 14, 15 + 16 and 17 + 18 (S2 Table), respectively. Additionally, a non-related gene from chromosome 28, encoding a putative ribose-5-phosphate isomerase B (*RPIB*, ~570 bp) was used as control, using primers 19 + 20 (S2 Table). For Southern-blot analysis, total genomic DNA was extracted. Ten μg of genomic DNA were digested O/N with a 5 fold excess of SacI and NdeI, at 37°C and samples were run O/N in an agarose gel. The gel was sequentially incubated with 0.25 M HCl, 1.5M NaCl 0.5M NaOH and 3M NaCl 0.5M Tris.HCl pH 7. DNA was then transferred O/N onto a Nylon membrane (Amersham), using 10x SSC (saline sodium citrate: 300 mM sodium citrate, 1 M NaCl). Nucleic acids fixation was achieved at 65°C for 5 hours. Hybridization and revelation were undertaken using Gene Images AlkPhos Direct Labelling and Detection System kit (GE Healthcare Amersham). Pre-hybridisation, hybridisation and washes took place at 65°C, probe labelling and membrane stripping were performed according to the manufacturer instructions. The blots were probed sequentially with 5’ UTR, *LiASA*, *HYG* and *NEO*, which were PCR amplified, using primers 1 + 2, 9 + 10, 7 + 8 and 5 + 6 (S2 Table), respectively.

### *In vitro* growth of *LiASA* mutants

Cultures were launched and monitored microscopically every 24h for 8 days or were maintained in log phase by subculturing every 2 days and cumulative growth was assessed for 5 consecutive passages. The growth experiments were performed in complete RPMI (cRPMI) or Asn depleted cRPMI (cRPMI + L-asparaginase) obtained by cRPMI O/N incubation with 1250 U/L of L-asparaginase at 37°C. Growth curves were also undertaken in a serum-free RPMI (sfRPMI [46]) incubated with L-asparaginase, that was removed afterwards by flowing the medium through a 3 kDa Millipore centrifugal filter (Amicon Ultra), generating an Asn free medium (sfRPMI + L-asparaginase). Asn was directly added to the Asn free sfRPMI (cf = 380 μM) to generate complemented sfRPMI (sfRPMI + L-Asparaginase + Asn). Finally, growth curves were performed in complete M199 (cM199) [29] or cM199 supplemented with Asn (cf = 380 μM). Growth curves of *LiASA* mutants and WT were seeded at 1 x 10^6^ parasites/ml at 26°C, except in the case of sfRPMI, whose initial parasite density was 2 x 10^6^ parasites/ml, grown with agitation. Before launching growth curves, the parasites were maintained in log phase for 2–3 passages in the absence of selection drugs.

### *In vivo* infectivity of *LiASA* mutants

Five to six weeks old female BALB/c mice were obtained from Charles River. For each mouse injection, 1 x 10^8^ promastigotes recovered from 4 days old stationary culture were washed, resuspended in PBS, and injected intraperitoneally. Mice of each group (n = 4) were sacrificed at 2 weeks post-infection. The parasite burden in the spleen and liver was determined by limiting dilution as previously described [47].

### Digitonin fractionation

For each sample condition, 1 x 10^8^ promastigotes were washed once with cold trypanosome homogenisation buffer (THB), composed by 25 mM Tris, 1 mM EDTA and 10% sucrose, pH 7.8. Just before cell lyses, peptidase inhibitor (Roche) and different digitonin (Calbiochem) quantities (final concentrations of 12.5, 25, 50, 100, 200, 500 and 1000 μg/ml) were added to 250 μl of cold THB, for cell pellet resuspension. Untreated cells and those completely permeabilised (total release, the result of incubation in 1% Triton X-100) were used as controls. Each sample was incubated 60 min on ice, and then centrifuged at 13,000 rpm, 4°C, for 10 min. Supernatants were taken off into new pre-cold tubes and 250 μl of cold THB was added to each pellet and then mixed. All fractions were analysed by WB.

### Immunofluorescence

*L*. *infantum* mid-log promastigotes were fixed, permeabilised and stained as previously described [48]. Cells were incubated with primary antibody O/N at 4°C. The following primary antibodies were used: rabbit anti-*Li*AS-A (1:1000) and sheep anti-*Li*TDR1 (thiol-dependent reductase 1, 1:2000). Subsequently, slides were incubated for 1h at RT in a dark humidified atmosphere with a secondary antibody (1:500). The following secondary antibodies were used: goat anti-rabbit Alexa Fluor 488 or 568 and donkey anti-sheep Alexa Fluor 488 (Molecular probes, Life Technologies). In the case of Mitotracker Orange (Invitrogen), we stained the parasites by adding 1 μM to culture medium (without FBS) for 1h at 26°C, prior to the above described procedure. Slides were stained and mounted with Vectashield-DAPI (Vector Laboratories, Inc.). Images were captured using fluorescence microscope AxioImager Z1 (Carl Zeiss), equipped with a Axiocam MR v. 3.0 camera (Carl Zeiss), using either 63x (Plan-Apochromat 63x/1.40 Oil DIC) or 100x (Plan-Apochromat 100x/1.40 Oil DIC) objective. Images analysis and deconvolution was performed using ImageJ software (v. 1.47) and image deconvolution lab plugin (2010 Biomedical Imaging Group, EPFL, Switzerland) with Richardson-Lucy algorithm.

### Statistical analysis

For statistical analysis, one-way ANOVA and two-tailed Student’s test were used. Statistical analysis was performed using GraphPad Prism Software (version 5.0): statistical significance *p* ˂ 0.05 (*), *p* ˂ 0.01 (**), *p* ˂ 0.001 (***), *p* ˂ 0.0001 (****).

## Results

### *Li*AS-A and *Lm*AS-A sequence alignment and homology models

**Fig 1:**
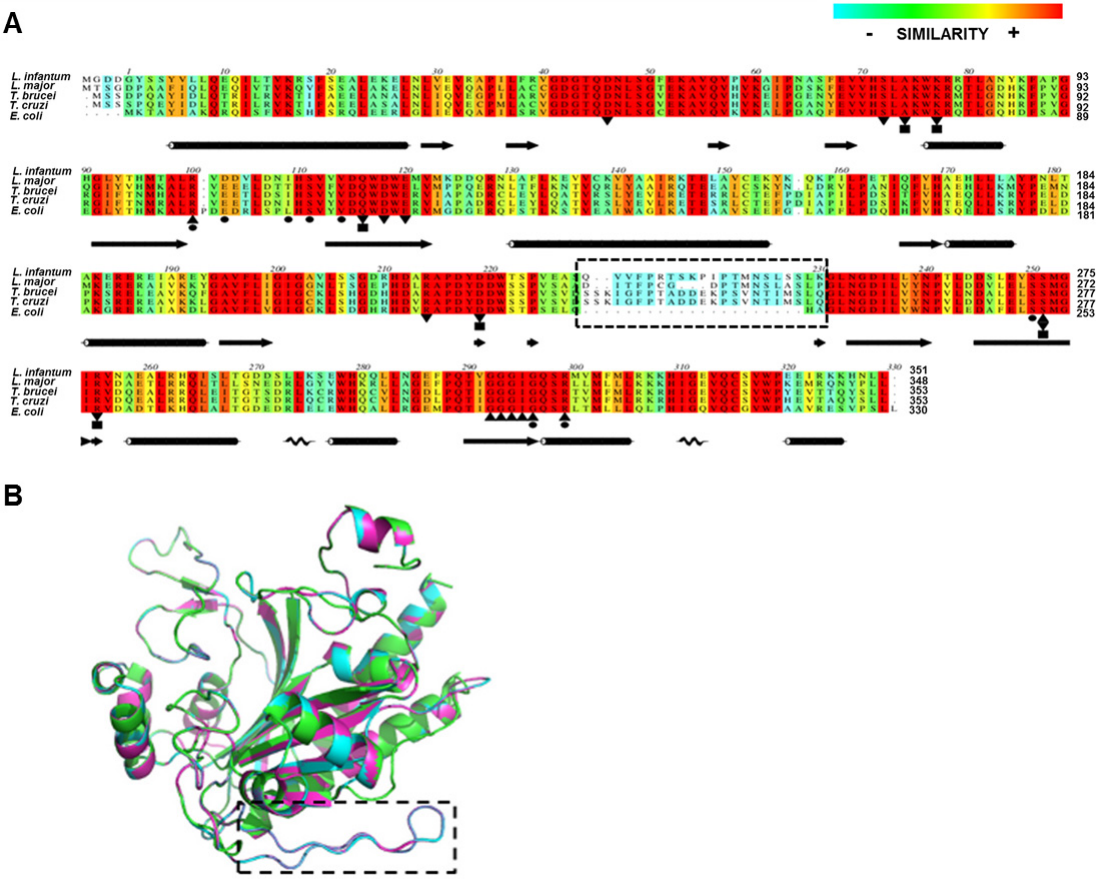
Multiple-sequence alignment of prokaryote and eukaryote AS-A proteins and 3D homology models of *Li*AS-A and *Lm*AS-A. **A)** Alignment of *Li*AS-A (NCBI-Gene ID: 5069795/ LinJ.26.0790), *Lm*AS-A (NCBI-Gene ID: 5652811/LmjF.26.0830), *Tb*AS-A (NCBI-GeneID:3658321/ Tb927.7.1110), *Tc*AS-A (NCBI-GeneID:3534325/Tc00.1047053503625.10) and *Ec*AS-A (NCBI-GeneID:948258/pdb:12AS). A pre-established colour pattern was used, according to ALSCRIPT Calcons (Aline version 011208): red, identical residues; orange to blue, scale of conservation of amino acid properties in each column alignment; white, dissimilar residues). Secondary structure components of *Ec*AS-A crystal structure (black) are represented above the alignment. In all sequences, binding residues for several ligands were represented: AMP (circles), asparagine (squares), ATP (triangle) and aspartate (inverted triangle). **B)** Superposition of *Ec*AS-A structure (green) (PDB accession code 12AS), with *Li*AS-A (blue) and *Lm*AS-A (purple) homology models (obtained from the SWISS-MODEL server, using PDB 12AS as a template). The dashed box points a structurally divergent region.

The open reading frames (ORFs) encoding putative AS-A and AS-B enzymes were identified in the genomes of *L*. *infantum* JPCM5 (LinJ.26.0790; LinJ.29.1590) and *L*. *major* Friedlin (LmjF.26.0830; LmjF.29.1490) [42–44]. The *ASA* amplified sequence from *L*. *infantum* strain matched 100% the annotated sequence from JPCM5 genome. To obtain structural and functional insights on AS-A enzymes, we have performed *in silico* analysis using the *L*. *infantum* (*Li*AS-A), *L*. *major* (*Lm*AS-A), *T*. *brucei* (*Tb*AS-A), *T*. *cruzi* (*Tc*AS-A) and *E*. *coli* (*Ec*AS-A) sequences that generate polypeptides containing 353, 353, 351, 348 and 330 residues, respectively (Fig 1A). Overall, the sequence alignment shows a high conservation of the main structural features, including the active site residues (Fig 1A). Indeed, the amino acids involved in Asn binding are strictly conserved across species, whereas in the case of AMP binding pocket, the majority of residues are conserved with a few exceptions. For instance, in the case of *Li*AS-A and *Lm*AS-A, there is a sole residue replacement, namely *Ec*AS-A L109, corresponding to I111 in both cases. This residue is not involved in polar interactions with AMP molecule, but instead integrates the outer wall of the nucleotide binding pocket [15].

Analysing the homology models of *Li*AS-A and *Lm*AS-A (Fig 1B) obtained by superimposition with *Ec*AS-A crystal structure (PDB 12AS [15]), there is a divergent region highlighted with a dashed rectangle (Fig 1B) in *L*. *infantum* and *L*. *major* enzymes, which is strictly conserved in these two species. This region also exists in trypanosomes, although little conservation is found when comparing to *Leishmania* sp. (Fig 1A, [28, 29]).

### Enzymatic characterization of *Li*AS-A

**Fig 2:**
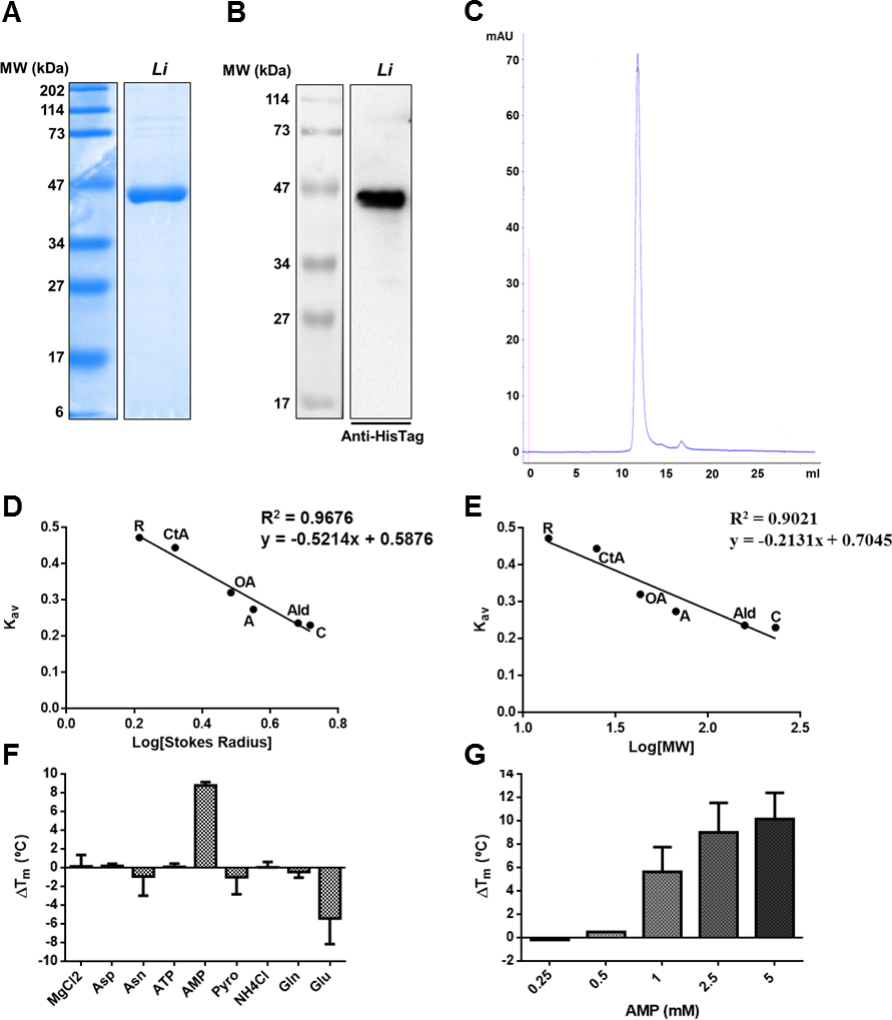
Analysis of recombinant *Li*AS-A. **A)** Coomassie blue stained 12% SDS-PAGE gel of 10 μg of recombinant *Li*AS-A post affinity chromatography purification. **B)** Western-blot analysis of 1 μg of purified recombinant *Li*AS-A using a rabbit anti-HisTag monoclonal antibody (1:1000). MW, molecular weight marker. **C)** Analytic size exclusion chromatogram of recombinant *Li*AS-A after purification by affinity, size exclusion and ion exchange chromatographies. **D and E)** Calibration curve for *Li*ASA Stokes’ radius and MW determination, respectively. K_av_ was determined considering the elution volume of the proteins used as standards, the total volume of the column and the exclusion volume given by the elution of blue dextran. The used standards were as follows: ribonuclease (R), chymotrypsinogen A (CtA), ovalbumin (OA), albumin (A), aldolase (Ald), catalase (C). Data is representative of two independent experiments. **F and G)** Differential scanning fluorimetry analysis of recombinant *Li*AS-A in the presence of several ligands, expressed in T_m_ variation (∆T_m_ - °C) determined as T_m_ (protein + ligand)–T_m_ (protein without ligand). **F)** Single ligand effect at 1 mM concentration: ATP, AMP, pyrophosphate (Pyro), ammonium chloride (NH_4_Cl), magnesium chloride (MgCl_2_), asparagine (Asn), aspartate (Asp), glutamate (Glu), glutamine (Gln). **G)** Concentration dependent effect of AMP in *Li*AS-A stabilization. These results represent the mean values of two independent experiments plus the standard deviation.

Recombinant *Li*AS-A, comprising a 6 histidine N-terminal tag, was expressed in *E*. *coli* and purified by affinity chromatography in native conditions in order to evaluate and characterize its enzymatic activity. The protein presented the expected MW for the monomer, ~42 kDa, as presented on Fig 2A and 2B, with either Coomassie staining or Western-blot analysis with an anti-HisTag antibody, respectively. Subsequently, *Li*AS-A was further purified sequentially by size exclusion and ion exchange chromatographies, and the final fractions were analysed by analytic size exclusion chromatography (Fig 2C). Using the latter chromatography and calibration standards, Stokes’ radius (~3.52 nm, Fig 2D) and MW (~78.8 kDa, Fig 2E) were extrapolated in the protein native state. *Li*AS-A corresponds to a homodimer, as predicted.

For the characterization of the enzymatic activity of recombinant *Li*AS-A, a specific colorimetric assay that quantifies Asn formation was used [28, 45]. The optimal pH for the enzymatic activity was 7.6. The kinetic characterization of the enzyme was undertaken in steady-state conditions, using a fixed concentration of 8.4 mM of Mg^2+^ (Table 1). *Li*AS-A displayed ammonia and glutamine dependent activity

**Table 1:** Kinetic parameters of *Li*AS-A for aspartate, ATP, ammonia and glutamine.

Substrate	*L*. *infantum*		
	*K*_m_ (mM)	*k*_cat_ (s^-1^)	*K*_sp_* (M^-1^.s^-1^)
**Aspartate**	6.21 ± 1.15	9.19 ± 0.69	1.48 x 10^3^
**ATP**	1.47 ± 0.04	4.18 ± 0.02	2.84 x 10^3^
*Ammonia dependent activity*			
**Ammonia**	1.12 ± 0.16	7.46 ± 0.33	6.66 x 10^3^
*Glutamine dependent activity*			
**Glutamine**	1.71 ± 0.28	4.51 ± 0.19	2.64 x 10^3^

*Specificity Constant (*k*_cat_ / *K*_m_)

The values are means ± standard deviations obtained from 3 independent experiments.

When comparing *K*_m_ values for ammonia and glutamine, no statistical significant difference is found (*p* = 0.03), but there is significance in the differences found in *k*_cat_ (*p* = 1.80 x 10^−4^). In order to discard the possibility that utilization of glutamine as a substrate was an artefact resulting from contamination with *Ec*AS-B (*Ec*AS-B ~120 kDa), highly purified fractions of *Li*AS-A (*Li*AS-A ~84 kDa) were tested and glutamine utilization were clear in all protein samples tested.

Differential scanning fluorimetry was also used in order to further understand the relevance of the different substrates for thermal stabilization of the enzyme. AS-A forms a crucial β-AspartylAMP-Mg^2+^ intermediate, which then undergoes a nucleophilic attack of ammonia, forming Asn and releasing AMP e pyrophosphate [15]. According to our data, ammonia can be free or glutamine-derived, although the glutaminase domain of *Li*AS-A remains to be identified. Looking at the differential scanning fluorimetry data, AMP leads to a 10 degrees shift in *Li*AS-A T_m_, thermally stabilising this protein (Fig 2F) in a concentration dependent fashion (Fig 2G).

### *LiASA* null mutants generation by targeted gene replacement

**Fig 3:**
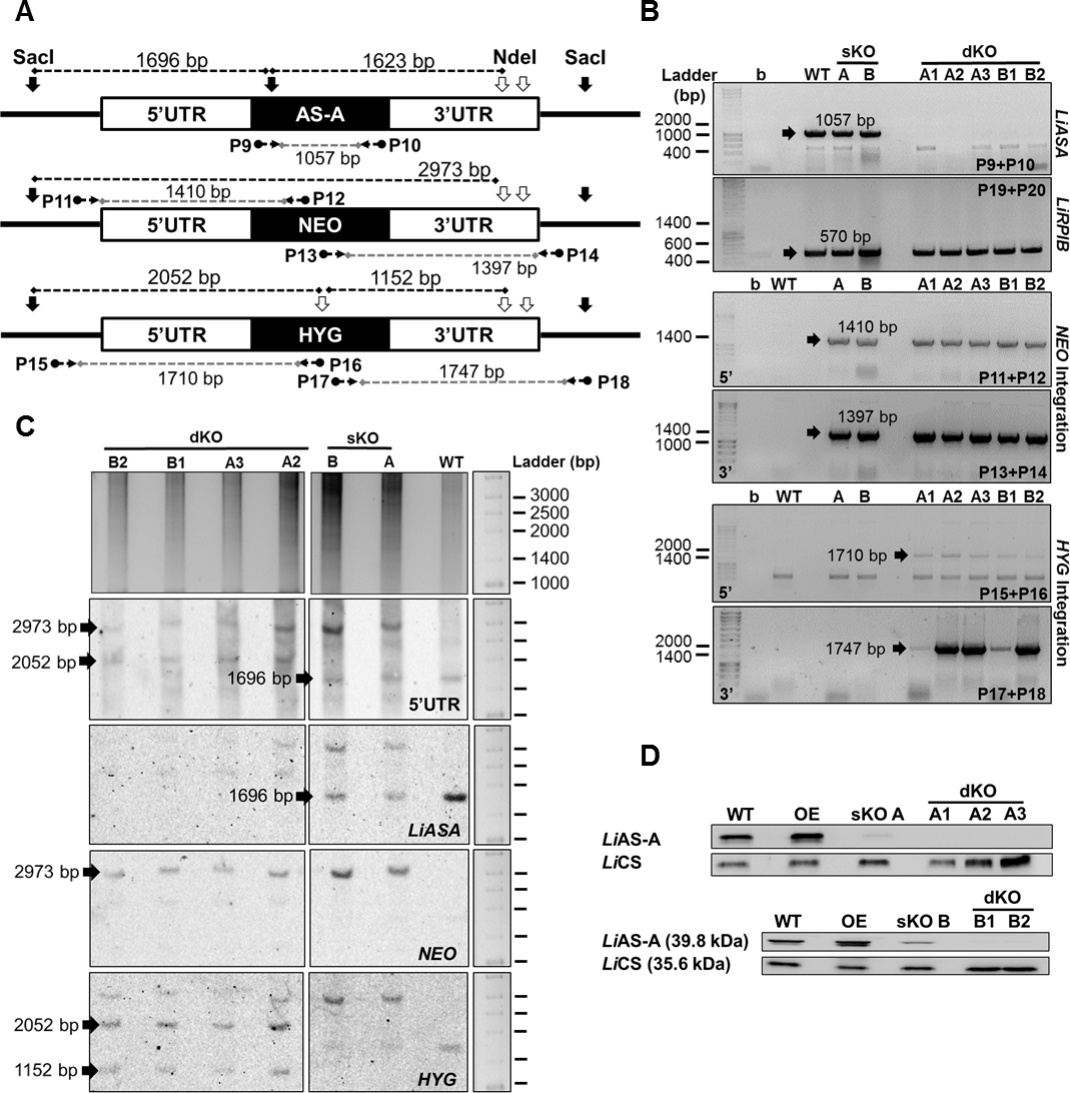
Genetic and post-translational analysis of the *LiASA* mutants. **A)** *LiASA* locus schematics: *ASA* allele and targeted gene replacement cassettes, containing *NEO* and *HYG* resistance genes. Horizontal black arrows and numbers represent the primer pairs used to assess the genotype of the mutants: the grey dashed line represents the expected PCR fragment. Southern-blot approach, upon digestion with NdeI (vertical black contoured arrows) and SacI (vertical black full coloured arrows) is also represented: dashed black lines represent the expected digestion fragments. **B)** PCR analysis of *LiASA* mutants to assess *LiASA* presence, *NEO* 5’ and 3’ integration and *HYG* 5’ and 3’ integration. Additionally, non-related *LiRPIB* gene from chromosome 28 was amplified as a control. b, blank. **C)** Southern-blot analysis of 10 μg of *LiASA* mutants (*versus* WT) genomic DNA, previously digested with NdeI and SacI, and probed using 5’ UTR. Subsequently, the blot was stripped and reprobed 3 additional times, using *LiASA*, *NEO* and *HYG*. **D)** Western-blot analysis of *Li*AS-A expression in promastigote mutants (*versus* WT) using *Li*CS (cysteine synthase) as loading control. OE, overexpressor.

A targeted gene replacement strategy was used for inactivation of the *ASA* gene of *L*. *infantum*. Two constructs, obtained by fusion PCR, linking *NEO* or *HYG* to the 5’ and 3’ UTRs of the *LiASA* gene were used to remove the first and second *LiASA* allele, respectively. Two sKO mutants (clones A and B) were transfected with the *HYG* construct. We successfully obtained 5 dKO mutants, 3 from clone A (A1, A2 and A3) and 2 from clone B (B1 and B2). The integration of the resistance markers in the expected locus was confirmed by PCR using primers upstream of the 5’ UTR or downstream of the 3’UTR coupled with primers in their ORFs (the strategy is illustrated on Fig 3A). *NEO* 5’ and 3’ integration was positive in both sKO and dKO mutants, as for *HYG*, only in dKO parasites, as expected (Fig 3B). Also by PCR analysis, we could not amplify *LiASA* ORF in null mutants (a non-related gene–*LiRPIB*- was amplified as control–Fig 3B).

Southern-blot analysis confirmed the genotypes: the expected fragments upon digestion with SacI and NdeI are represented on Fig 3A. A first hybridisation was performed using 5’ UTR as a probe: in WT a single band of ~1696 bp corresponding to *LiASA* was generated, with twice the intensity observed in the sKO mutants that possess a single copy, and absent in the dKO mutants, confirming the successful gene removal (Fig 3C). In both sKO and dKO clones, a band of ~2973 bp was generated corresponding to *NEO*, and then only in dKO clones, a band of ~2052 bp corresponding to *HYG* was observed (Fig 3C). The blot was then stripped and reprobed three additional times to confirm each one of the bands (faint bands of incomplete stripping can be observed), sequentially using *LiASA*, *NEO* and *HYG*. All the mutants were also analysed by Western-blot, showing a protein reduction in sKO mutants and a complete absence in the dKO clones (Fig 3D). *LiASA* gene was cloned into a pSP72*αBLASTα* vector in order to obtain an overexpressor mutant (OE) as well (Fig 3D).

### AS-A is localized in the cytosol of *L*. *infantum* promastigotes

Rabbit polyclonal antibodies produced against recombinant *Li*AS-A recognised a major band in total WT promastigotes extract with the expected molecular weight (~39.8 kDa [web.expasy.org/protparam], S1A Fig), but not in a dKO mutant (dKO A2). Prior to immunolocalisation studies, *Li*AS-A antibody was also validated (S1B Fig) by performing an IFA and comparing the labelling intensity in WT promastigotes *versus LiASA* null mutants and OE. A positive correlation between protein level and fluorescence intensity was found on WT *versus* OE (S1C Fig). As expected, no specific labelling was detected for the *Li*AS-A null mutants (S1B and S1C Fig).

**Fig 4:**
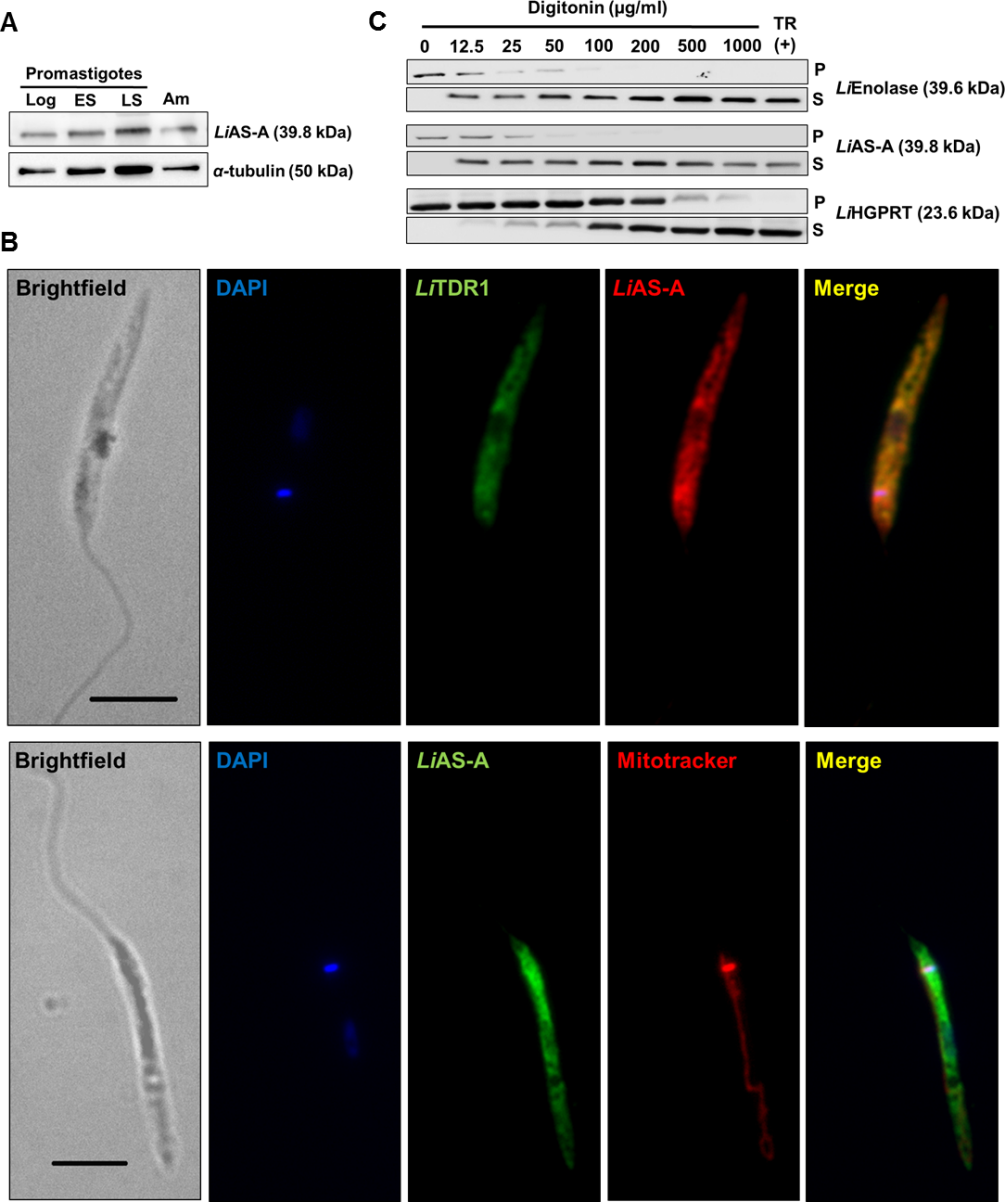
*Li*AS-A expression and localization in *L*. *infantum*. **A)** AS-A expression in different stages of *L*. *infantum* life cycle. Promastigote forms: logarithmic phase (Log), early stationary phase (ES), late stationary phase (LS); axenic amastigote forms (Am). Twenty μg of total extract were analysed by Western-blot and probed with rabbit polyclonal anti-*Li*AS-A. α-tubulin (mouse monoclonal antibody) was used as loading control. These results are representative of 3 independent experiments. **B)** Immunofluorescence analysis showing AS-A (red upper panel; green lower panel) localization in *L*. *infantum* promastigote form. Nucleus and kinetoplast DNA, cytosol and mitochondria were stained with DAPI (blue), sheep anti-*Li*TDR1 (thiol-dependent reductase in green) and Mitotracker Orange CMTMROS (red), respectively. Images were acquired with a 100x objective, using a Zeiss AxioImager Z1. The scale bar corresponds to 5 μm. Data is representative of 4 independent experiments. **C)** Digitonin fractionation of mid-log *L*. *infantum* promastigotes. Pellet (P) and supernatant (S) fractions obtained using increasing concentrations of digitonin or positive control with 1% of Triton X-100 (TR–Total Release) were subjected to Western-blot analysis and probed with antibodies against *Li*Enolase (cytosolic marker) and hypoxanthine guanine phosphoribosyltransferase *Li*HGPRT (glycosomal marker). Data is representative of 5 independent experiments.

Using α−tubulin (~50 kDa) as loading control we compared the expression levels of *Li*AS-A in different developmental stages: promastigotes (logarithmic, early stationary and late stationary phase) and axenic amastigotes (Fig 4A). No significant differences were observed.

Immunofluorescence analysis showed that in promastigotes *Li*AS-A co-localises with *Li*TDR1 (thiol-dependent reductase 1), which is a cytosolic protein involved in thiol metabolism [49] (Fig 4B, upper panel). *Li*AS-A subcellular localisation in promastigotes was also assessed by digitonin fractionation. The fractioning profile was evaluated using antibodies for proteins present in different subcellular compartments, namely, anti-*Tb*Enolase (*Li*Enolase *versus Tb*Enolase 79% identity, *Li*Enolase 39.6 kDa) as cytosolic marker [50], and anti-*Ld*HGPRT (hypoxanthine guanine phosphoribosyltransferase, 23.6 kDa) as glycosomal marker [51]. *Li*Enolase (Fig 4C) can be found in the supernatant for digitonin concentrations as low as 12.5 μg/ml, and retained in the pellets up to 25–50 μg/ml. *Li*HGPRT (Fig 4C), which localises to the glycosomes, is detected in the supernatant in appreciable amounts for higher digitonin concentrations and is retained longer in the pellet (up to 200–500 μg/ml, and residually at 1000 μg/ml of digitonin). As expected, *Li*AS-A presents a profile similar to *Li*Enolase, supporting a cytosolic location (Fig 4C).

Intriguingly, *Ld*AS-A was reported to have dual localisation between the cytoplasm and mitochondria in promastigote form [29]. Due to the high identity (~99%) between both enzymes, we investigated whether *Li*AS-A also localised to the mitochondria. Immunofluorescence analysis of *Li*AS-A subcellular distribution on promastigotes labelled with mitotracker showed no evidence of mitochondrial location (Fig 4B, lower panel). Moreover, by using tools for protein localisation prediction (TargetP, CELLO, MITOPROT and Predotar), mitochondria localisation seems unlikely and actually, CELLO predicts cytoplasmic localisation. In conclusion, our data shows *Li*AS-A localises to the cytosol.

### *Li*AS-A is required for promastigotes growth only in asparagine limiting conditions

**Fig 5:**
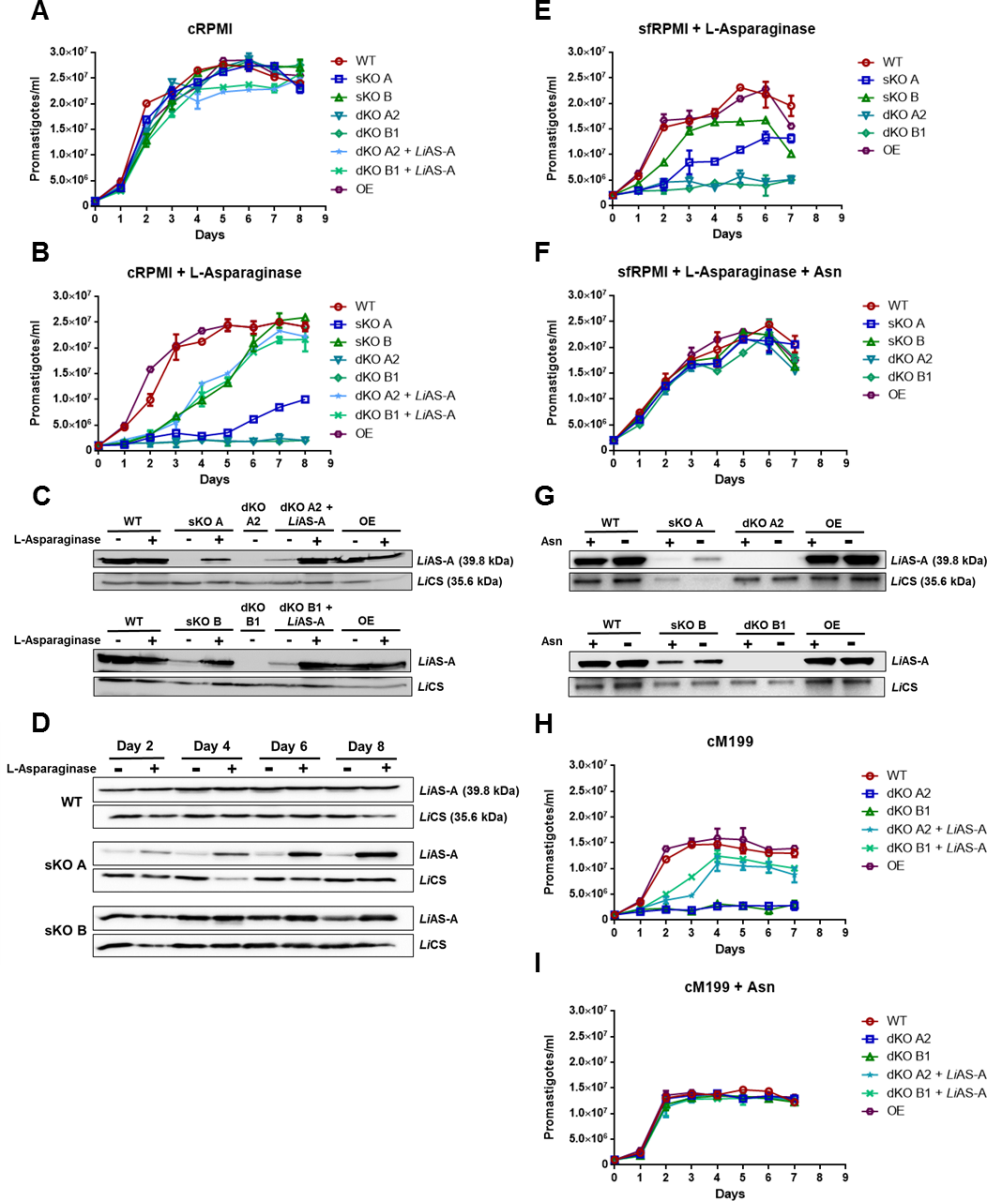
*In vitro* growth of *LiASA* mutants in normal or Asn depleted medium and respective *Li*AS-A expression levels. ** A and B)** *L*. *infantum* promastigote growth curves in cRPMI or cRPMI + L-asparaginase, respectively, including dKO mutants (clones A2 and B1) complemented with pSPα*BLAST*α carrying *LiASA* gene. In B, a significant growth difference in comparison to WT (*p* ˂ 0.05, Graphpad Prism 5.0 version) was found for sKO A, sKO B, dKO A2 and B1, dKO A2 + *Li*AS-A, dKO B1 + *Li*AS-A and OE mutants for days 1 to 8, days 1 to 6, days 1 to 8, days 2 to 6, days 2 to 7 and day 3, respectively. **C)** Western-blot analysis of *Li*AS-A expression in 7 days old *LiASA* mutants (*versus* WT) cultured in cRPMI and cRPMI + L-asparaginase. **D)** Western-blot analysis of *Li*AS-A expression levels over time in WT, and clones A and B cultured in cRPMI (lane L-asparaginase -) or cRPMI + L-asparaginase (lane L-asparaginase +). **E and F)** *L*. *infantum* promastigote growth curves in sfRPMI + L-asparaginase or sfRPMI + L-asparaginase + Asn, respectively. sfRPMI was Asn depleted through incubation with L-asparaginase, which was then removed by flowing the medium through an Amicon Column of 3 kDa pore (sfRPMI + L-asparaginase). Asn was then added directly to the medium (sfRPMI + L-asparaginase + Asn). In E, a significant growth difference in comparison to WT (*p* ˂ 0.05, Graphpad Prism 5.0 version) was found for sKO A, sKO B and dKO A2 and B1 mutants for days 2 to 7, days 2 and 5 to 7 and days 2 to 7, respectively. **G)** Western-blot analysis of *Li*AS-A expression in 7 days old *LiASA* mutants (*versus* WT) cultured in sfRPMI + L-asparaginase (lane Asn -) and sfRPMI + L-asparaginase + Asn (lane Asn +). **H and I)** *L*. *infantum* promastigote growth curves in cM199 and cM199 + Asn, respectively, including dKO mutants complemented with pSPα*BLAST*α carrying *LiASA* gene. In H, a significant growth difference in comparison to WT (*p* ˂ 0.05, Graphpad Prism 5.0 version) was found for dKO A2 and B1, dKO A2 + *Li*AS-A and dKO B1 + *Li*AS-A mutants for days 1 to 7, days 2 and 3, days 1 to 3, respectively. The results (A-I) are representative of 2 independent experiments. For the Western-blot analysis displayed in C, D and G, 1x10^7^ parasites were used for total extract preparation and *Li*CS (cysteine synthase) was used as loading control. OE, overexpressor.

All mutants displayed similar growth patterns comparing to WT promastigotes in cRPMI (Fig 5A). However, in Asn depleted medium, achieved upon L-asparaginase treatment (cRPMI + L-asparaginase), the behaviour was quite different for some of the mutants. Parasites overexpressing *Li*AS-A displayed a significant higher growth during log phase when comparing to the WT, whereas the dKO mutants (clones A2 and B1) displayed a major growth defect (Fig 5B). The complementation of these null mutants with an episome (pSP72α*BLAST*α) carrying *LiASA* gene rescued the growth (Fig 5B). Moreover, an upregulation in *Li*AS-A levels could be observed in these mutants in Asn limiting conditions (Fig 5C). Western-blot analysis also showed that in the same conditions, an upregulation in *Li*AS-A could also be observed over time in the sKO parasites (clones A and B), enabling the growth recovery in these mutants (Fig 5B–5D and S3 Table). This recovery was faster in sKO clone B that had higher basal levels of *Li*AS-A than clone A (Fig 5B and 5C). We also evaluated the cumulative growth under constant multiplicative conditions, in which high amino acids levels are required. For that, parasites were maintained in log phase in Asn replete or Asn depleting conditions, and the same patterns were observed (S2 Fig).

To ensure the defective growth phenotype of sKO and dKO parasites in L-asparaginase treated medium was due to Asn depletion, we supplemented this medium with Asn. Surprisingly the addition of this amino acid to L-asparaginase treated RPMI medium fails to reverse the observed growth delay/arrest phenotype. The fact that L-asparaginase was not inactivated or neutralized, and consequently may have remained active, may explain this result. Consequently, we used another strategy by undertaking growth curves in a serum free medium (sfRPMI [46]) incubated with L-asparaginase that was removed afterwards using a 3 kDa Amicon column. In sfRPMI devoid of Asn (sfRPMI + L-asparaginase), the same growth defect of the sKO and dKO mutants was observed (Fig 5E). And then again, in the sKO clones the upregulation of *Li*AS-A allowed the growth rescue (Fig 5G and S3 Table). When adding back Asn (sfRPMI + L-Asparaginase + Asn), all mutants grew in a similar fashion (Fig 5F). In the absence of drug pressure and in normal conditions, parasites provided of an episome carrying *LiASA* (OE) hardly overexpress AS-A, however, under Asn depleting conditions, they upregulate its expression (comparing to the levels in the WT, there is an increase from ~130% to ~300% and from ~110 to ~130%, in panels C and G, respectively, and S3 Table).

Moreover, besides the experiments using L-asparaginase treatment, we have also performed growth curves in a medium formally lacking Asn–complete M199 (cM199)—in order to further confirm Asn auxotrophy upon *ASA* ablation. In this medium, null mutants presented a growth defect comparable to the one observed in cRPMI + L-asparaginase, which again was reversed when these mutants were complemented with an ectopic copy of *ASA* gene (Fig 5B *versus* 5H). The addition of Asn to the final concentration of 380 μM (like in RPMI) rescues the growth defect displayed by the null mutants in cM199 (Fig 5I). Interestingly, the experiments to assess *Li*AS-A essentiality in *L*. *donovani* were performed in cM199 [29], therefore the inability to generate *LdASA* null mutants may be due to the performance of those attempts in Asn limiting conditions.

In conclusion, *ASA* deletion renders parasites auxotrophic to Asn, but is dispensable for parasite growth in normal conditions.

### *In vivo* infectivity of *LiASA* mutants

**Fig 6:**
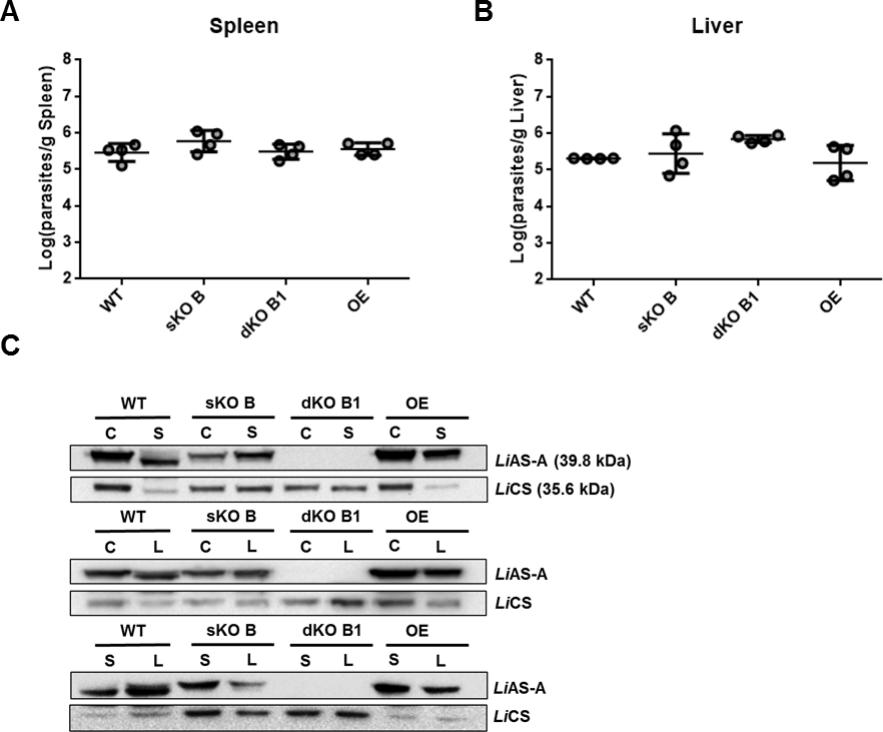
*In vivo* infectivity of *LiASA* mutants in mice. **A and B)** Stationary promastigotes were intra-peritoneally injected in BALB/c mice that were sacrificed 2 weeks post infection, in order to determine parasite burden in spleen (A) and liver (B). The values represent the means of four independent animals ± standard deviation **C)** Western-blot analysis of *Li*AS-A levels in WT and *LiASA* mutants: parasites maintained in culture (C) comparing with parasites recovered from spleen (S) or liver (L). The data is representative of 2 independent experiments carried out with 2 different clones for each genotype. For the Western-blot analysis displayed in C, 1x10^7^ parasites were used for total extract preparation and *Li*CS (cysteine synthetase) was used as loading control. OE, overexpressor.

Notwithstanding, we intended to evaluate the impact of *LiASA* ablation on, *in vivo* infectivity. Five to six old female BALB/c mice were infected and were sacrificed at 2 weeks post-infection. The parasite burden in the spleen (Fig 6A) and liver (Fig 6B) was not statistically different in *LiASA* mutants when compared to the WT. The same scenario was observed for sKO A and dKO A2 mutants. No differences in *Li*AS-A expression levels were found when comparing parasites used in mice infection (Culture) to parasites recovered from spleen (S) or liver (L) (Fig 6C). Thus, *Li*AS-A ablation does not compromise parasite infectivity in the context of an acute *in vivo* infection.

## Discussion

Despite being eukaryotes, trypanosomatids, present AS-A enzymes of bacterial origin. Moreover, these enzymes are aminoacyl-tRNA synthetase paralogues, displaying an AsnRS catalytic core with conserved class II motifs, yet lacking the tRNA binding domain [27]. In this work, we have demonstrated that *Li*AS-A is able to synthesize Asn using either ammonia or glutamine as nitrogen donors, as previously described for *Tb*AS-A, *Tc*AS-A and *Ld*AS-A [28, 29]. *K*_m_ values for aspartate and ATP are close to the ones determined for *Tb*AS-A and *Tc*AS-A [27]. As for ammonia, the *K*_m_ value found for *Li*AS-A is 5 fold lower in comparison to *Tb*AS-A, *Tc*AS-A and *Ld*AS-A [27, 28]. In the case of *Ld*AS-A, the *K*_m_ values for aspartate were around 10 fold lower [29] than the ones obtained for *Li*AS-A. Regarding the high conservation of the active sites among *Leishmania* AS-A enzymes, we cannot exclude that the observed kinetic differences may be due to the differences in the amount of protein that is properly folded, especially taking into account they are expressed in a heterologous system. Moreover, it is important to emphasize that the kinetic determinations for *Ld*AS-A were performed using a different experimental set up. Importantly, *Tb*AS-A and *Tc*AS-A use preferably ammonia [28], whereas *Li*AS-A seems to use both roughly in the same extent (Table 1). AS-A activity in trypanosomatids more resembles AS-B enzymes, concerning both the optimal pH for enzymatic activity (7.6 instead of 8) and also the ability to use both nitrogen donors. AS-B enzymes use preferably glutamine, with exception of the human enzyme that presents approximately the same affinity for both nitrogen sources [18, 19, 25, 52–57]. This biochemical feature, so far only described for trypanosomatids AS-A enzymes [28, 29], becomes particularly interesting in the context of the presence of an ORF encoding a hypothetical, yet non-classical, AS-B, in the genome of these organisms (*L*. *infantum* [LinJ.29.1590], *L*. *major* [LmjF.29.1490], *T*. *brucei* [Tb927.3.4060] and *T*. *cruzi* [Tc00.1047053510001.40]) [42–44]. These sequences contain a Pfam AS domain (pfam00733) and glutamine hydrolysing domains in the C and N-terminus, respectively. BLASTp analysis of *L*. *infantum* sequence, for instance, revealed several hits that corresponded to hypothetical proteins from a broad range of eukaryotes. However, we have no evidence AS-B is functional at all.

Much remains to be disclosed regarding the AS-A enzymes from trypanosomatids, for instance, we still lack information on their glutamine binding and hydrolysing sites. *Tb*AS-A crystallisation only emphasised the high conservation of Asn and AMP binding pockets, as the only divergent region from *Ec*AS-A (a 19 residues insertion, also present in *Li*AS-A and *Lm*AS-A, Fig 1) was not visible in the experimental electron density maps and therefore likely disordered [29]. This insertion displays little conservation when comparing *Leishmania* and trypanosomes, and its role on a structural or functional level is still unclear.

AS-A is a key enzyme in Asn metabolism that was proposed as a potential drug target due to its absence in the human host. Moreover, AS-A was reported to be essential for *L*. *donovani* survival, contrasting with *T*. *brucei* bloodstream forms, as in the latter it was shown to be dispensable for both *in vitro* growth and infectivity. These findings pointed to a differently regulated Asn homeostasis across trypanosomatids. In *L*. *infantum*, our efforts to generate *ASA* null mutants were successful, indicating the gene is not essential for survival. Moreover, the null mutants did not present any growth or infectivity defect. Our *in vitro* growth data demonstrate that upon *LiASA* deletion, promastigotes become dependent on extracellular Asn for optimal growth (Fig 5). These results suggest that even if AS-B is functional, it does not compensate *Li*AS-A activity, as *LiASA* null mutants fail to grow in Asn limiting conditions. Additionally, WT parasites grew normally in Asn depleted medium without AS-A upregulation, suggesting Asn synthesis by basal AS-A suffices the cellular needs, although the mutants overexpressing this enzyme had a metabolic advantage in an Asn deprivation environment during log phase (S2B and S2E Fig). We can actually infer the parasite can both synthesise and take up this amino acid, and the latter fully compensates the former. Furthermore, our results indicate that *Li*AS-A levels are regulated according to Asn availability, and it was equally surprising to see how fast and efficiently sKO mutants were able to upregulate AS-A when cultured in Asn limiting conditions (Fig 5D). It is also noteworthy that the two sKO mutants displayed a substantial difference in AS-A levels, which has also been observed among other sKO mutants generated in this study. A possible explanation might be that the two allelic copies may differently affect *ASA* expression.

In trypanosomatids, much remains to be unravelled concerning amino acid transporters (AATs) and mostly the pathways involved in amino acid sensing and regulation of their synthesis and uptake [34]. Very few data is available in the literature concerning Asn transport in these parasites. In *T*. *brucei*, a protein presenting putative orthologues in *Leishmania* [42–44] was characterized as a transporter of several neutral amino acids, including asparagine (*Tb*AATP1) [58]. In mammalian cells, AS-B is a transcriptional target of the well characterized GCN2/elF2α/ATF4 axis, in response to amino acid starvation [59, 60]. The phosphorylation of elF2 leads to a repression of general protein synthesis, as well as an activation of gene-specific translation. In *Saccharomyces cerevisiae*, GCN2, which is activated by amino acid, glucose or purine deprivation, is the only elF2 kinase, contrasting with mammals that possess some additional three, HRI, PKR and PEK/PERK [61]. *T*. *brucei* and *L*. *donovani* PERK orthologues [62, 63] have been implicated in the response to ER stress and their activation leads to a decrease in the overall translation [62]. At the moment, it is still not clear whether phosphorylation of elF2 in trypanosomatids would result in a downstream signalling cascade, as bZIP type transcription factors, that could act like GCN4 or ATF4, are absent in these organisms [64].

The close relation between *L*. *infantum* and *L*. *donovani* species and the 99% identity of AS-A between both makes the discrepant phenotype intriguing. In the literature, several cases in which knocking out a gene can have different impact on virulence depending on the species can be found. [65]. Nevertheless, to our knowledge, there is no documented example among cutaneous or among visceral species of a gene that is detrimental for survival in one species and dispensable in other closely related species. However, we did find a case of differences at a strain level for instance [66]. Nonetheless, firstly we must highlight that *LdASA* essentiality was claimed solely based on the consecutive failure in the removal of the second gene copy [29]. Secondly, our results suggest that the medium in which the experiments were performed, cM199, may explain this difference. The former lacks Asn and *LiASA* null mutants could not grow unless upon Asn supplementation (Fig 5H and 5I). These results reinforce the importance of the medium composition when attempting gene knock-out of metabolic enzymes, and supplementation may be detrimental when potentially generating auxotrophs [67, 68].

*LiASA* dKO mutants displayed no compromised infectivity in mice, suggesting that in intracellular amastigote form, either AS-B is functional or, most likely, parasites are able to uptake Asn in such an extent that compensates the lack of intracellular synthesis. *In vivo* treatment with L-asparaginase, which induces a decrease in Asn bloodstream levels, has been successfully used for years in the treatment of acute lymphoblastic leukemia [69] and recently it was proposed as a promising strategy to treat bacteremia caused by group A *Streptococcus* and eventually other extracellular bacteria [13]. However, if for some extracellular pathogens, L-asparaginase treatment seems promising, in the case of an obligate intracellular microorganism, even when simultaneously inhibiting the microbial AS-A, several issues may arise, namely the potential contribution of the host cell for Asn *de novo* synthesis.

Taken all together, we conclude AS-A is not a suitable drug target candidate in *L*. *infantum*, and therefore, with regard to drug development, such a protein target becomes pointless against *Leishmania*.

## Supporting Information

S1 Table Oligonucleotides sequences used to obtain *Li*AS-A recombinant protein (P1-P2) and pSPα*BLAST*α*LiASA* (P3-P4). (DOCX)

S2 Table Oligonucleotides sequences used to obtain gene replacement cassettes (P1-P8) and to confirm *LiASA* mutants’ genotype (P9-P20). (DOCX)

S3 Table Semi-quantification of AS-A levels in *LiASA* mutants cultured *in vitro* in Asn replete or depleting conditions. (DOCX)

S1 Fig Rabbit polyclonal anti-*Li*AS-A antibody validation. **A)** Western-blot analysis of WT and *LiASA* null mutant (clone A2) promastigotes extracts using rabbit polyclonal anti-*Li*AS-A (1:1000). **B)** Representative immunofluorescence images of different genotypes (WT, dKO clone A2 and OE) of mid-log *L*. *infantum* promastigotes, using rabbit polyclonal anti-*Li*AS-A antibody (1:1000). Upper and lower panels present brightfield and *Li*AS-A (green) + DAPI (blue) stained images, respectively. Images were acquired with a 100x objective, using a Zeiss AxioImager Z1. The scale bar corresponds to 5 μm. **C)** Fluorescence intensity quantification in WT, dKO clone A2 and OE parasites when stained with anti-*Li*AS-A antibody (1:1000). The values are expressed in CTCF (corrected total cell fluorescence), and background (BG) values are displayed as well. The quantification was performed on images acquired with 63x objective, using a Zeiss AxioImager Z1 and the same exposure time for all genotypes (*Li*AS-A 400 ms; DAPI 100 ms). Twenty different fields for each genotype were analysed in duplicate, and the fluorescence of an average of 50–100 parasites was quantified using ImageJ (v 1.47) software. Statistical analysis was performed using Graphpad Prism 5.0 version: statistical significance *p* ˂ 0.05 (*), *p* ˂ 0.01 (**), *p* ˂ 0.001 (***), *p* ˂ 0.0001 (****). The results (A-C) are representative of 2 independent experiments.
(TIFF)

S2 Fig Cumulative *in vitro* growth of logarithmic *LiASA* mutants in normal or Asn depleted medium. **A/D and B/E)** *L*. *infantum* promastigotes growth curves of *LiASA* mutants (*versus* WT), cultured in cRPMI and cRPMI + L-asparaginase, respectively. Parasites were maintained in logarithmic phase by subculturing every 2 days. The results correspond to mean values of duplicates ± standard deviation. Statistical analysis was performed using Graphpad Prism 5.0 version: statistical significance *p* ˂ 0.05 (*), *p* ˂ 0.01 (**), *p* ˂ 0.001 (***), *p* ˂ 0.0001 (****). **C and F)** Western-blot analysis of *Li*AS-A expression levels in 8 days old promastigotes, cultured in cRPMI and cRPMI + L-asparaginase. In A-F panels, the results are representative of two independent experiments. For the Western-blot analysis displayed in C and F, 1x10^7^ parasites were used for total extract preparation and *Li*CS (cysteine synthase) was used as loading control.
(TIFF)
