# Supplementary material for: Leishmania infantum Asparagine Synthetase A Is Dispensable for Parasites Survival and Infectivity
Source: PLoS Negl Trop Dis. 2016 Jan 15;10(1):e0004365. doi: 10.1371/journal.pntd.0004365 (PMC4714757; doi:10.1371/journal.pntd.0004365)
Supplement: S1 Table — (DOCX) [file pntd.0004365.s001.docx]

| **Primer Sequence** |
| --- |
| **1** 5' CAATTTGCATATGTCGTCCAGTCCGCAG 3'  **2** 5' CCCAAGCGAATTCTTACAATAAAGAGTAC 3'  **3** 5' GTCTAGAATGTCGTCCAGTCCGCAGGAGTACATTGA 3'  **4** 5' GCATATGTTACAATAAAGAGTACTGCGCCGTGACC 3' |

**Table S1.** Oligonucleotides sequences used to obtain *Li*AS-A recombinant protein (P1-P2) and pSPα*BLAST*α*LiASA* (P3-P4)
