# Supplementary material for: Leishmania infantum Asparagine Synthetase A Is Dispensable for Parasites Survival and Infectivity
Source: PLoS Negl Trop Dis. 2016 Jan 15;10(1):e0004365. doi: 10.1371/journal.pntd.0004365 (PMC4714757; doi:10.1371/journal.pntd.0004365)
Supplement: S2 Table — (DOCX) [file pntd.0004365.s002.docx]

| **Primer**  **Sequence** |
| --- |
| **1** 5' CAGCCTGGAGGAGAACATTG 3'  **2** 5' GGGATGAATGGAGGGGTGTTG 3'  **3** 5' CCACAAAATGCCAGGGAGAAG 3'  **4** 5' GTAATCGTCCACGCCAGAAG 3'  **5** 5' CCTTTTATTCAACACCCCTCCATTCATCCCATGATTGAACAAGATGGATT 3'  **6** 5' CTGCAGAGAGCTTCTCCCTGGCATTTTGTGGTCAGAAGAACTCGTCAAGAAGGCGATAG 3'  **7** 5' GTCGCCAAGCCCTTTTATTCAACACCCCTCCATTCATCCCATGAAAAAGCCTGAACTCAC 3'  **8** 5' CTGCAGAGAGCTTCTCCCTGGCATTTTGTGGCTATTCCTTTGCCCTCGGACGAGTG 3'  **9** 5' TCGTCCAGTCCGCAGGAGTACA 3'  **10** 5' ACAATAAAGAGTACTGCGCCGTGACC 3'  **11** 5' CGGGTCCACGATTCACTGGAAG 3'  **12** 5' CAGGTAGCCGGATCAAGCGTATGC 3'  **13** 5' CGAGCACGTACTCGGATGGAAG 3'  **14** 5' GACCTCAACAGGAGCAACCTAAAG 3'  **15** 5' AACTTTATGCGCAGCGTCAC 3'  **16** 5' GGCCCAAAGCATCAGCTCATC 3'  **17** 5' GCAGGCTCTCGATGAGCTGATG 3'  **18** 5' AGGAAGGAAACACGCAAAGGTC 3'  **19**  5' ATGCCGAAGCGTGTTGCTCTG 3'  **20** 5' TTAAGACCTCCACAACCGCTGAAG 3' |

**Table S2.** Oligonucleotides sequences used to obtain gene replacement cassettes (P1-P8) and to confirm *LiASA* mutants’ genotype (P9-P20)
