# Supplementary material for: Leishmania infantum Asparagine Synthetase A Is Dispensable for Parasites Survival and Infectivity
Source: PLoS Negl Trop Dis. 2016 Jan 15;10(1):e0004365. doi: 10.1371/journal.pntd.0004365 (PMC4714757; doi:10.1371/journal.pntd.0004365)
Supplement: S3 Table — (DOCX) [file pntd.0004365.s003.docx]

| **Mutants** | **L-Asn** | **cRPMI(C)** | **sfRPMI(G)** |
| --- | --- | --- | --- |
| **sKO A** | **+** | 0.05 | 0.01 |
|  | **-** | 0.68 | 1.77 |
| **sKO B** | **+** | 0.66 | 0.52 |
|  | **-** | 0.93 | 0.99 |
| **dKO A2** | **+** | SBG | SBG |
|  | **-** | ND | SBG |
| **dKO B1** | **+** | SBG | SBG |
|  | **-** | ND | SBG |
| **dKO A2 + *Li*AS-A** | **+** | 0.25 | ND |
|  | **-** | 1.12 | ND |
| **dKO B1 + *Li*AS-A** | **+** | 0.41 | ND |
|  | **-** | 1.07 | ND |
| **OE** | **+** | 1.30 | 1.10 |
|  | **-** | 3.02 | 1.34 |

**Table S3.** *Li*AS-A levels quantification in *LiASA* mutants in *in vitro* Asn replete or depleting conditions

Results were normalized against *Li*CS (cysteine synthase) and are expressed in protein ratio against WT parasites cultivated in Asn non depleting conditions

Results in C and G correspond to the Western-blots depicted in these panels of the figure 5 (in the case of OE, the values correspond to the mean of 2 independent blots).

SBG – Similar to the background

ND – Non determined
