# Supplementary figures and images for: Leishmania infantum Asparagine Synthetase A Is Dispensable for Parasites Survival and Infectivity
Source: PLoS Negl Trop Dis. 2016 Jan 15;10(1):e0004365. doi: 10.1371/journal.pntd.0004365 (PMC4714757; doi:10.1371/journal.pntd.0004365)

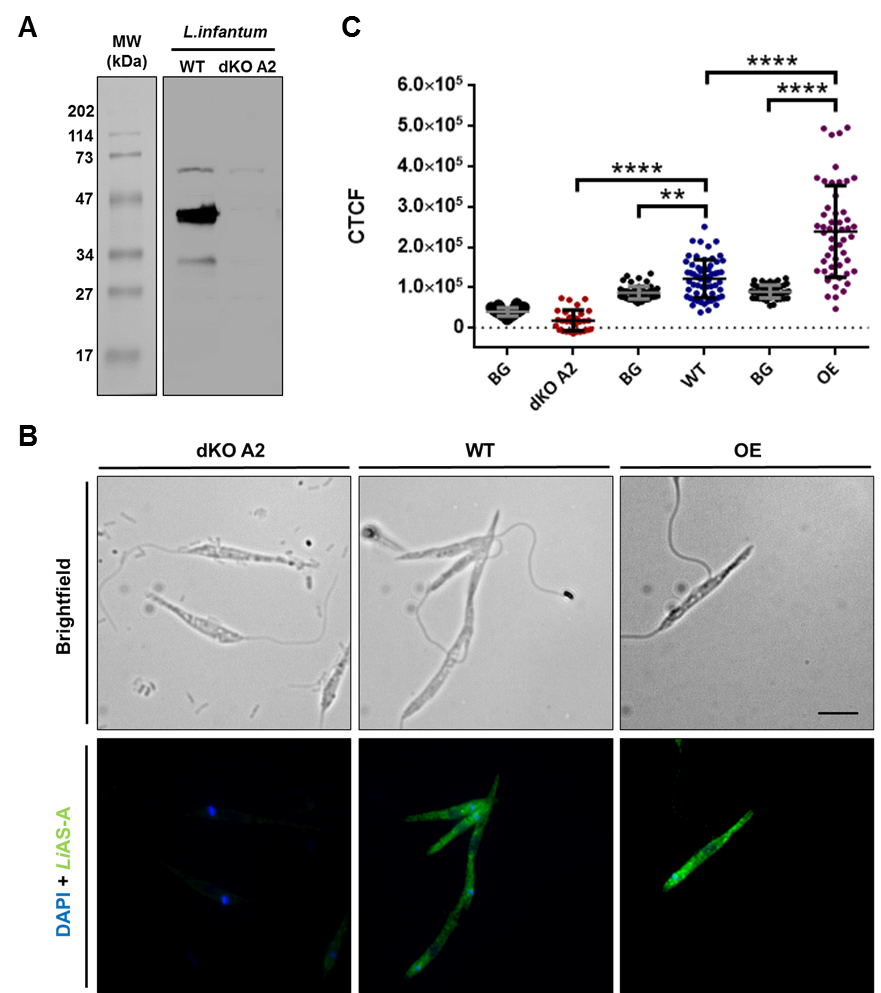

Supplement: S1 Fig — A) Western-blot analysis of WT and LiASA null mutant (clone A2) promastigotes extracts using rabbit polyclonal anti-LiAS-A (1:1000). B) Representative immunofluorescence images of different genotypes (WT, dKO clone A2 and OE) of mid-log L. infantum promastigotes, using rabbit polyclonal anti-LiAS-A antibody (1:1000). Upper and lower panels present brightfield and LiAS-A (green) + DAPI (blue) stained images, respectively. Images were acquired with a 100x objective, using a Zeiss AxioImager Z1. The scale bar corresponds to 5 μm. C) Fluorescence intensity quantification in WT, dKO clone A2 and OE parasites when stained with anti-LiAS-A antibody (1:1000). The values are expressed in CTCF (corrected total cell fluorescence), and background (BG) values are displayed as well. The quantification was performed on images acquired with 63x objective, using a Zeiss AxioImager Z1 and the same exposure time for all genotypes (LiAS-A 400 ms; DAPI 100 ms). Twenty different fields for each genotype were analysed in duplicate, and the fluorescence of an average of 50–100 parasites was quantified using ImageJ (v 1.47) software. Statistical analysis was performed using Graphpad Prism 5.0 version: statistical significance p ˂ 0.05 (*), p ˂ 0.01 (**), p ˂ 0.001 (***), p ˂ 0.0001 (****). The results (A-C) are representative of 2 independent experiments. (TIFF) [file pntd.0004365.s004.tiff]

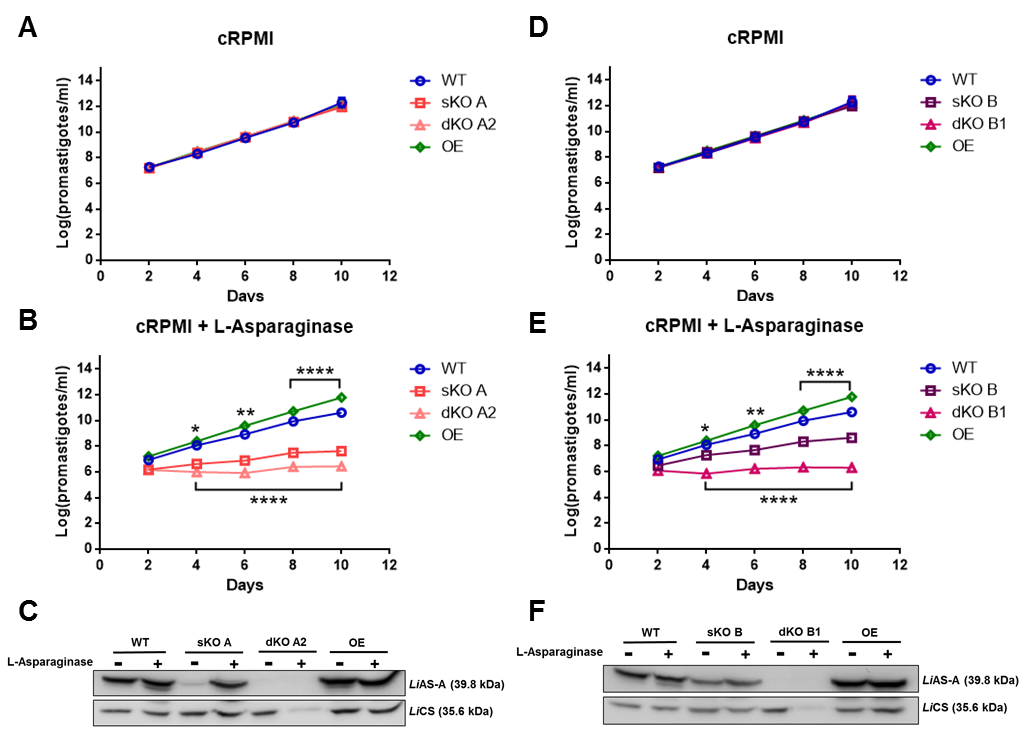

Supplement: S2 Fig — A/D and B/E) L. infantum promastigotes growth curves of LiASA mutants (versus WT), cultured in cRPMI and cRPMI + L-asparaginase, respectively. Parasites were maintained in logarithmic phase by subculturing every 2 days. The results correspond to mean values of duplicates ± standard deviation. Statistical analysis was performed using Graphpad Prism 5.0 version: statistical significance p ˂ 0.05 (*), p ˂ 0.01 (**), p ˂ 0.001 (***), p ˂ 0.0001 (****). C and F) Western-blot analysis of LiAS-A expression levels in 8 days old promastigotes, cultured in cRPMI and cRPMI + L-asparaginase. In A-F panels, the results are representative of two independent experiments. For the Western-blot analysis displayed in C and F, 1x10^7^ parasites were used for total extract preparation and LiCS (cysteine synthase) was used as loading control. (TIFF) [file pntd.0004365.s005.tiff]
